# Eco-Friendly Carbon Fiber Woven Fabric-Based Sound Absorbers with Tunable Air Cavity Structures for Advanced Noise Control

**DOI:** 10.1021/acsomega.5c10560

**Published:** 2026-01-27

**Authors:** Jung-Hwan Oh, Yong-Won Kwon

**Affiliations:** National Center for Carbon Neutral Technology Strategy, 140841 Korea Institute of Energy Research (KIER), 152 Gajeong-ro, Yuseong-gu, Daejeon 34129, Republic of Korea; Department of Advanced Materials, 732598 Research Institute of Medium & Small Shipbuilding (RIMS), 38-6 Noksansandan 232-ro, Kangseo-gu, Busan 46757, Republic of Korea

## Abstract

This study introduces an eco-friendly class of carbon fiber woven fabric composites (CFFCs) incorporating tunable air cavity architectures to achieve lightweight broadband acoustic absorption. By systematically varying the layer number, fiber orientation, and cavity configuration, we establish a clear structure–property–performance relationship that governs resonance tuning, viscous–thermal dissipation, and air-flow tortuosity. Acute angle stacking enhances in-plane shear deformation and frictional damping, producing up to ∼3 dB improvement in layer-to-layer impact noise attenuation, as confirmed through interfacial impact tests. Incorporation of single or multiple air cavity units further shifts the resonance to lower frequencies and achieves high absorption levels (SA*C*_max_ ≤ 0.99) within an ultrathin (<7 mm) profile. Full system impact noise transmission measurements in a two-story house model demonstrate up to ∼35 dB reduction in transmitted sound pressure, verifying the practical applicability of cavity-integrated CFFCs as lightweight noise control elements. Overall, this work provides a scalable and sustainable materials platform that leverages woven anisotropy and cavity-induced resonances to deliver tunable broadband acoustic performance in next-generation composite systems.

## Graphical abstract

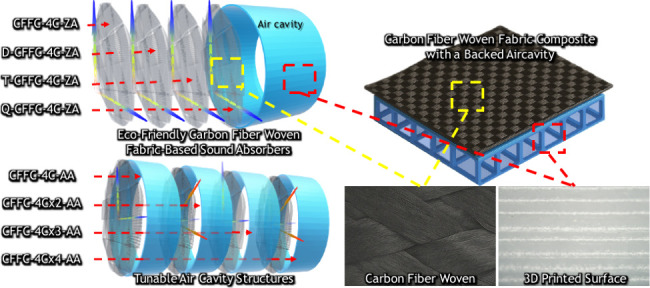

## 1 Introduction

Noise pollution has emerged as a significant public health and environmental challenge, increasing the demand for high-performance sound-absorbing materials. Conventional porous absorbers typically require substantial thickness to achieve effective mid- to high-frequency absorption, and their performance often deteriorates when the pores become contaminated.^1^ Panel-type absorbers can target lower frequencies, but their resonant behavior depends strongly on plate mass and panel-to-wall distance, creating undesirable trade-offs in weight and installation volume.^2^ Carbon fiber woven fabrics have gained attention as lightweight structural materials with potential acoustic functionality owing to their intrinsic porosity, flexibility, and tunable architecture. Their interyarn pores and undulated weave promote viscous and thermal dissipation when interacting with incident sound waves, making them attractive for multifunctional composite design.^3,4^ However, achieving broadband and low-frequency sound absorption with thin and lightweight structures remains challenging. Existing approaches using porous foams or resonant panels struggle to simultaneously satisfy performance, durability, and thickness constraints. A design strategy that couples the microscale dissipation of a woven fabric with macroscale cavity-induced resonance offers a promising route to overcome these limitations.^5–13^

Herein, to overcome these trade-offs, we propose a carbon fiber woven fabric-based composite (CFFC) integrating tunable air cavity structures. We hypothesize that the synergistic combination of cross-layered carbon fiber orientation and tunable air-cavity geometry enables broadband, low-frequency sound absorption while maintaining mechanical integrity and eco-friendly manufacturability.^14,15^ The objective of this study is to systematically design and evaluate these carbon fiber woven fabric-based composites to establish a scalable and sustainable platform for advanced noise-control applications.

## 2 Methodology/Experimental

### 2.1 Material Design and Fabrication Strategy

A commercially available 3K carbon fiber twill fabric (Keunyung, Korea) was used to fabricate the carbon fiber woven fabric-based composites (CFFCs). The fabric exhibited a mass per unit area of 117 g m^–2^ and an apparent porosity of 75%, as determined from the specimen dimensions and mass measurements. To prepare the CFFC layers, the fabric was laminated from one to four plies and stacked either in a zero angle (ZA, 0°) alignment following the twill direction or in an acute angle (AA, 45°) orientation. All specimens were prepared under consistent tensioning, trimming, and alignment conditions to ensure reproducibility. The cavity layer was constructed using 3D printed polylactic acid (PLA) rings (thickness: 0.4 mm) with cavity heights of 4 or 6 mm. The PLA rings were fabricated using FDM printing (XY-2, TRONXY) with the parameters such as layer thickness 0.20 mm, infill density 60%, printing speed 45 mm·s^–1^, nozzle temperature 210 °C, and bed temperature 60 °C. Single-cavity (CFFC-C) and multicavity (CFFC-CxN) configurations were assembled by alternating CFFC layers with PLA cavity rings. All abbreviations and symbols used in this study are summarized in Table 1 for ease of reference. The proposed composite is considered eco-friendly because it employs recyclable carbon fibers and biodegradable PLA, enabling sustainable and low emission fabrication.

**Table 1:** List of Abbreviations and Nomenclature

Abbreviation	Definition/Description
CFFC	Carbon Fiber Woven Fabric-Based Composite
ZA	Zero Angle (0° fiber orientation, aligned stacking)
AA	Acute Angle (±45° fiber orientation, cross-layered stacking)
CFFC-ZA	Carbon fiber woven fabric composite with Zero-Angle layer alignment
CFFC-AA	Carbon fiber woven fabric composite with Acute-Angle layer alignment
D-CFFC, T-CFFC, Q-CFFC	Double-, Triple-, and Quadruple-layer CFFC configurations
CFFC-C	CFFC with a single air cavity (backed structure)
CFFC-CxN	CFFC with multiple air cavities connected in series (*N* = number of cavities)
CFFC-4/6 - AA	Acute angle CFFC with air cavity thickness of 4 mm or 6 mm
CFFC-4/6 - ZA	Zero angle CFFC with air cavity thickness of 4 mm or 6 mm

### 2.2 Characterizations

The microstructure of the carbon fiber fabric was examined by using optical microscopy (Galaxy S22 Ultra) and scanning electron microscopy (Eclipse, Nikon). Tensile properties of the twill fabric were evaluated according to ASTM D3039 using a universal testing machine (AGX-V, Shimadzu) with a gauge width of 25 mm, gauge length of 250 mm, and a loading rate of 2 mm·min^–1^. In addition, specific surface area and porosity were characterized using a porometer (CFP-1100A, Porous Materials Inc.) following the ASTM F316 procedures.

Sound absorption measurements were performed using an impedance tube (SW260, BSWA) based on the two-microphone transfer-function method. All reported sound absorption coefficient (SAC) values correspond to normal incidence absorption based on ISO 10534–2. The 30 mm tube was used for 1,000 Hz to 6,100 Hz, and the 60 mm tube was used for 125–3,000 Hz. The two-microphone method inherently evaluates the complex reflection coefficient and the effective acoustic impedance of the specimen. Specimen thickness ranged from 0.26 to 25 mm, depending on the number of fabric layers and cavity depth. For each configuration, three separate specimens were prepared, and each specimen was measured at least three times. The SAC curves represent the averaged results of nine or more repeated measurements. All impedance tube measurements were conducted under controlled laboratory conditions (22 ± 2 °C, 40 ± 5% RH). Acoustic activity was subsequently calculated as the normalized integral of the sound absorption coefficient over the measurement range and was used as a broadband absorption index for comparing different configurations.

To evaluate impact noise reduction, a two-story acrylic house model (200 × 200 × 400 mm^3^) was constructed by using 3T acrylic plates. PLA cavity layers (4 mm or 6 mm) were 3D printed as 3 × 3 lattice grids and installed between floors. Carbon fiber layers (1–4 plies, ZA or AA) were placed above the cavity layer, and impact noise was induced by dropping a 19.4 g mass from the upper floor. Sound levels were recorded as Z-weighted RMS values in 1/3 octave bands using a real-time analyzer (SC202, CESVA).

## 3 Results and Discussion

Figure 1 summarizes the structural design space of the carbon fiber woven fabric composites (CFFCs) examined in this study. The configurations are classified into three categories based on key design variables: the number of carbon fiber fabric layers, the fiber orientation in each layer, and the presence and number of air cavities. CFFC specimens were fabricated with one to four layers, resulting in thicknesses ranging from approximately 0.27 to 1.08 mm, as shown in Figure 1a. Because layer count directly affects stiffness, mass, and structural rigidity, these configurations provide the fundamental baseline for understanding mechanical and acoustic trends discussed in later sections. The woven layers were aligned either in a zero angle (ZA, 0°) configuration following the twill direction or rotated by 45° (AA) to introduce in-plane anisotropy. These ZA/AA schemes were consistently applied to double-, triple-, and quadruple-layer stacks, enabling the controlled tuning of anisotropic properties and internal friction mechanisms relevant to acoustic behavior, as shown in Figure 1b. Air cavities were incorporated using 3D-printed PLA rings (4 mm or 6 mm height) to create single cavity (CFFC-C) or multicavity (CFFC-CxN) structures. Multiple cavities were formed by serially stacking CFFC-C units (N = 2–4), producing lightweight cavity-backed composites with increased design flexibility. These architectures enable systematic evaluation of cavity depth, number of resonant units, and their coupling with the woven microstructure. Together, the three categories in Figure 1 define the structural framework for the acoustic and mechanical analyses presented in the following sections.

**Figure 1:**
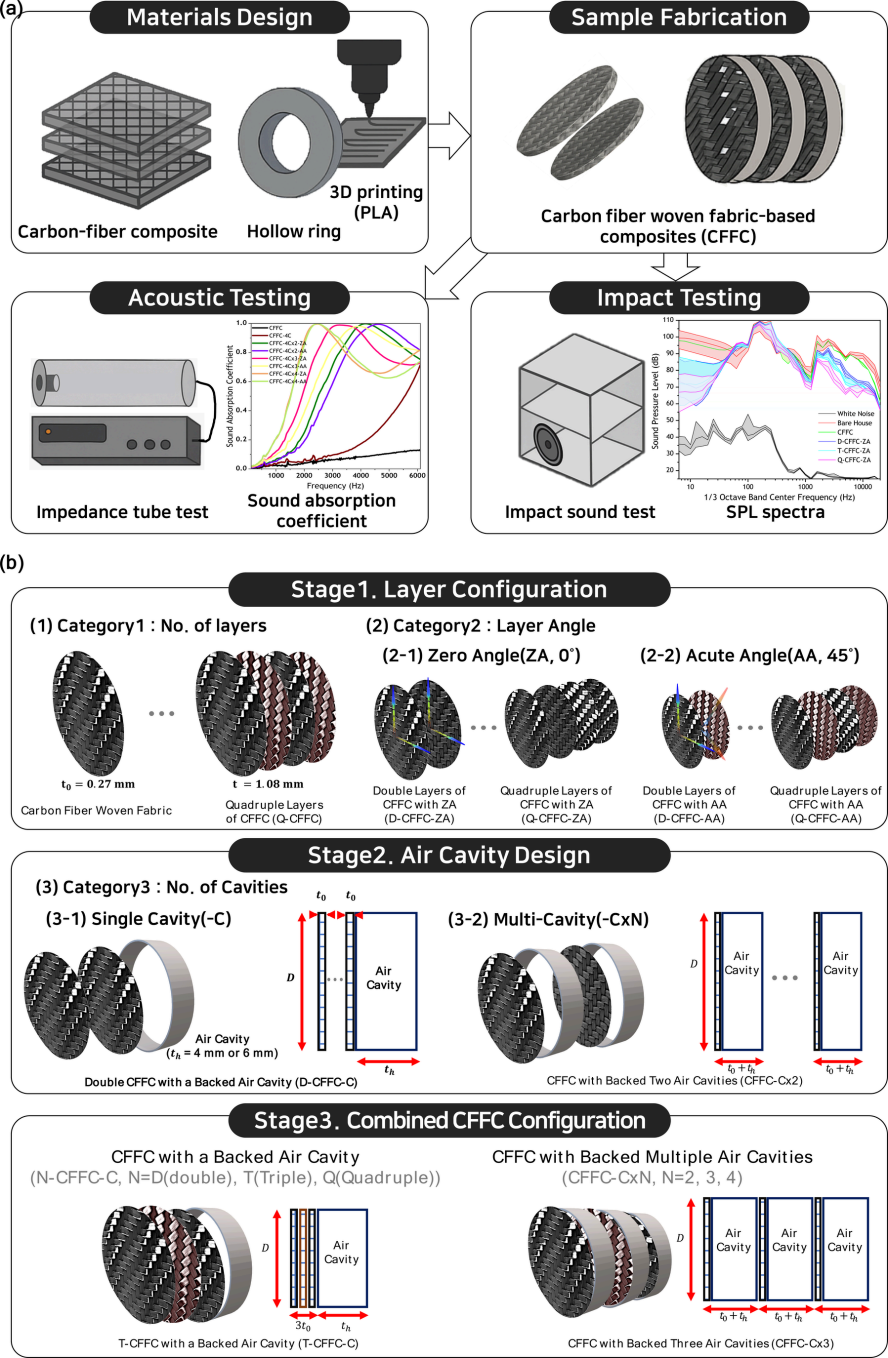
Design and configuration of carbon fiber woven fabric-based composites (CFFCs). (a) Schematic summary of the overall research workflow from materials design and fabrication to acoustic and impact testing illustrating the stepwise process for developing tunable sound absorbers. (b) Hierarchical classification of CFFC configurations according to design variables: Stage 1, layer configuration (number of layers and fiber orientation); Stage 2, air cavity design (single and multiple cavities); and Stage 3, combined hybrid structures integrating CFFCs with air cavities. This figure establishes the experimental framework and design hierarchy connecting composite architecture to the resulting acoustic performance.

To evaluate the sound absorption performance of carbon fiber woven fabric-based composites without air cavities, impedance tube measurements were conducted for the configurations shown in Figure 2. Figure 2a illustrates the experimental setup based on the two-microphone transfer function method. Representative CFFC specimens were mounted in the tube to examine the effects of the layer count and fiber orientation. Figure 2b and 2c shows the structural differences among single- to quadruple-layer stacks. Single-layer CFFC served as the baseline. For each layer count, specimens were fabricated in both zero angle (ZA, 0°) and acute angle (AA, ± 45°) configurations to identify the role of anisotropy on acoustic behavior. As shown in Figure 2d, the SAC spectra from 1,000 to 6,000 Hz reveal that up to triple-layer CFFC (T-CFFC), the ZA and AA orientations exhibit similar absorption characteristics. However, a clear divergence appears at the four layers (Q-CFFC). For Q-CFFC-ZA, the SAC shows a marked improvement at higher frequencies, reaching approximately 0.511 at around 5.9 kHz, compared to 0.418 for Q-CFFC-AA. This indicates that at higher layer counts, the ZA configuration becomes more effective at high-frequency absorption, likely due to reduced internal shear deformation and more uniform airflow pathways through the stacked weave. NRC values increase monotonically with layer count for both ZA and AA, confirming that greater thickness enhances broadband absorption, as shown in Figure 2e. Interestingly, for Q-CFFC, the zero angle configuration (Q-CFFC-ZA, NRC ≈ 0.039) exhibits a slightly higher NRC value compared to the acute angle configuration (Q-CFFC-AA, NRC ≈ 0.034). While the difference is small, the ZA configuration performs slightly better at high layer counts. Acoustic activity, defined as the normalized integral of SAC, follows similar trends and captures broadband absorption differences more clearly. Despite the thin overall thickness (<1.1 mm), incident sound can penetrate the interyarn pores of the woven fabric. Small vibrations of the flexible carbon filaments, combined with frictional and structural damping, enable energy dissipation. Orientation-dependent differences arise because ZA maintains a more uniform in-plane stiffness, whereas AA introduces anisotropic shear deformation. At higher layer counts, the more irregular internal geometry in AA may reduce effective pore pathways, slightly lowering high-frequency SAC. These results demonstrate that layer count is the dominant factor controlling broadband absorption in cavity free CFFCs, while fiber orientation provides secondary tuning of high-frequency response.

**Figure 2:**
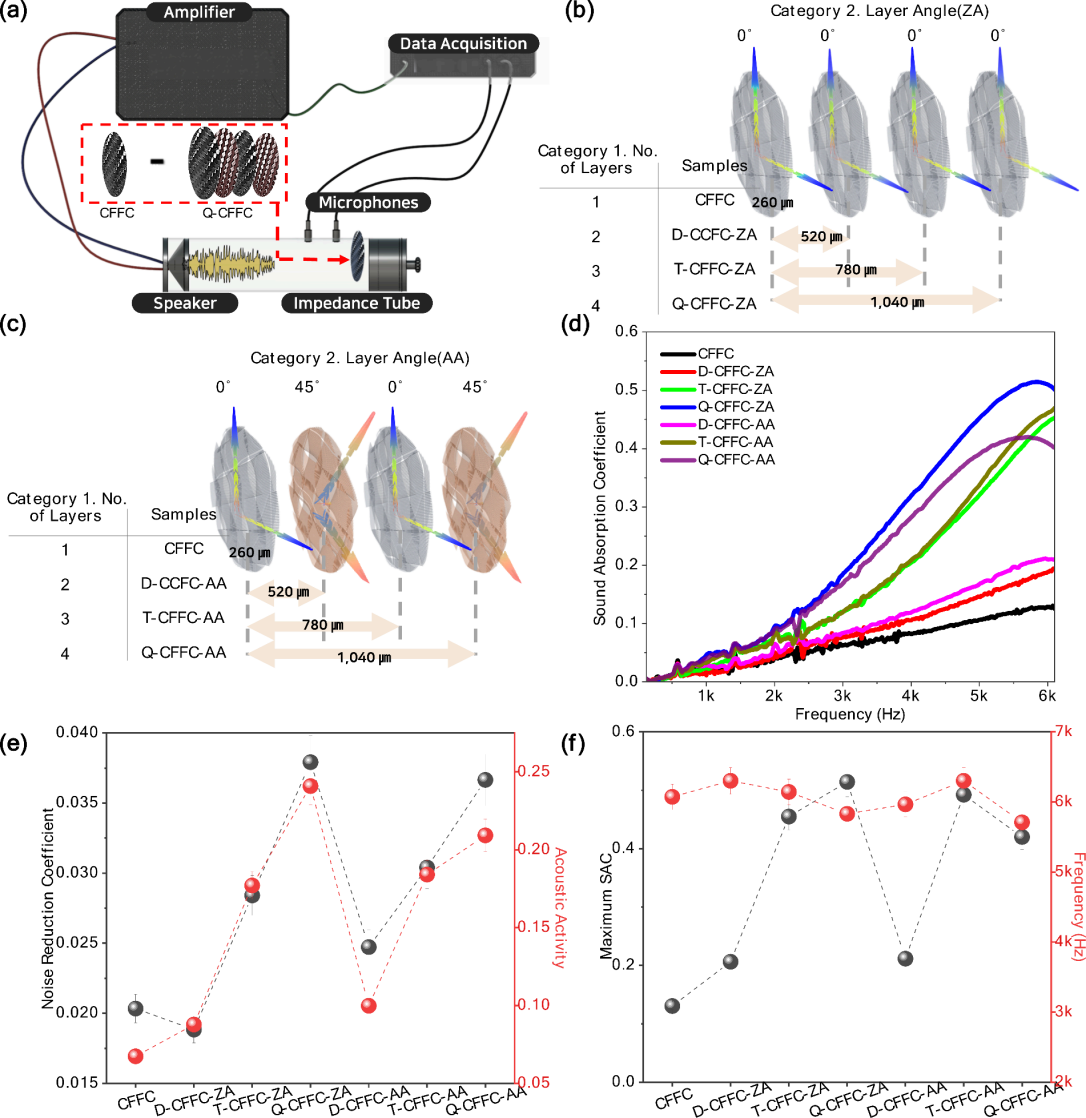
Acoustic performance of carbon fiber woven fabric-based composites (CFFCs) with varying layer configurations. (a) Schematic illustration of the impedance-tube setup used for sound-absorption measurements. 3D representations of the CFFC stacking structures with (b) ZA and (c) AA orientations for single- to quadruple-layer specimens. (d) Sound-absorption-coefficient (SAC) spectra as a function of frequency for different layer counts and fiber orientations. (e) Comparison of noise-reduction-coefficient (NRC) and acoustic-activity values. The results demonstrate that increasing the number of layers and controlling fiber orientation enhance broadband absorption and shift resonance peaks through internal friction and structural-damping effects, confirming the role of anisotropy in tuning acoustic response.

To examine the role of an internal air cavity, Figure 3 analyzes the sound absorption behavior of carbon fiber fabric composites incorporating a single cavity (CFFC-C). This extends the cavity-free baseline response of the CFFC specimens described in Figure 2. Figures 3a and 3e illustrate the stacking schemes for CFFC-C specimens, which follow the Category 1 and Category 2 principles defined in Figure 1, but introduce a hollow PLA ring (30 mm diameter, 0.4 mm thickness) to form the cavity. For the 4 mm cavity structures, total specimen height increases from 4.26 mm for single-layer CFFC-4C to 5.04 mm for quadruple-layer Q-CFFC-4C, with corresponding weights ranging from 0.34 to 0.82 g. For 6 mm cavities, specimen height ranges from 6.26 to 7.04 mm and weights range from 0.86 to 1.85 g. Full dimensional information is provided in Table S1. A clear and repeatable trend is observed in the NRC values (Figures 3c and 3g). For both cavity depths, transitioning from a single CFFC layer to a double-layer configuration produces a substantial increase in broadband absorption. In the 4 mm cavity set, the NRC rises from ∼ 0.03 for CFFC-4C to ∼ 0.15 for D-CFFC-4C-ZA and D-CFFC-4C-AA. Similarly, the NRC increases from 0.05 for CFFC-6C to ∼ 0.15 for D-CFFC-6C-ZA and D-CFFC-6C-AA in the 6 mm designs. This consistent NRC jump highlights a structural threshold: adding one additional carbon fiber layer above the cavity activates a strong cavity fabric coupling mechanism, transforming a weakly absorbing single layer system into a resonant two-layer absorber. Beyond the double layer configuration, the NRC increases nearly linearly with each additional layer, indicating predictable and incremental gains. As the number of woven layers increases, the reduced air permeability (Table S2) and more tortuous internal flow paths promote stronger viscous losses and multiple internal reflections. These effects become increasingly relevant at lower frequencies, where longer propagation paths assist in improving the absorption, particularly in cavity-backed configurations. Cavity thickness further modulates the acoustic activity. As shown in Figures 3c and 3g, acoustic activity for single layer specimens increases from ∼ 0.2 for the 4 mm cavity to ∼ 0.45 for the 6 mm cavity, confirming that deeper cavities enhance air structure oscillation energy even when only a single carbon fiber sheet is present. While NRC remains low for these single layer systems, the increase from 0.03 (4 mm) to 0.05 (6 mm) suggests that cavity enlargement provides a modest but measurable improvement in broadband absorption at a minimal layer count. Figures 3b and 3f present the SAC–frequency curves. For 4 mm cavities (Figure 3b), the dominant resonant peak lies between ∼ 3,687 and 5,612 Hz. Increasing the number of carbon fiber layers shifts the resonance toward lower frequencies, consistent with an increased areal density and stronger coupling with the cavity. Double layer structures exhibit sharply amplified absorption peaks: D-CFFC-4C-AA reaches SAC ≈ 0.99 at ∼ 5,612 Hz, and D-CFFC-4C-ZA reaches ∼ 0.94 at ∼ 5,372 Hz. This emergence of a strong mid- to-high-frequency resonance explains the dramatic NRC improvement observed when moving from one to two layers. The difference between the ZA and AA stacking configurations can be attributed to the anisotropic mechanical response of the woven fabric. The ± 45° orientation in AA introduces additional in-plane shear deformation and frictional damping between yarns, subtly modifying dissipation pathways and shifting resonance characteristics, effects that become more pronounced in cavity-backed architectures where small variations in interlayer deformation significantly influence the resonant response. Collectively, these results reveal a key design principle: a double layer carbon fiber fabric structure combined with a single air cavity represents the minimum effective configuration for achieving a step-change in broadband sound absorption. Increasing the cavity thickness inherently increases the internal void fraction of the composite, which may subtly influence its overall stiffness. Additional layers yield linear and predictable improvements, while increasing the cavity thickness provides a secondary parameter for enhancing acoustic activity, especially in low layer structures. For reference, our CFFC absorber was compared with recent composite-type systems, including 3D-printed lattices and polymer-textile cavity absorbers.^16,17^ Despite structural differences, the CFFC achieves α_max_ ≈ 0.95 at ≤ 6 mm, confirming high efficiency at a reduced thickness. The difference between ZA and AA configurations can be attributed to the anisotropic behavior of the woven fabric: the ± 45° layers in AA introduce additional in-plane shear deformation and frictional damping, which alter the dissipation pathway and modify the resonance response, especially in cavity-backed structures.

**Figure 3:**
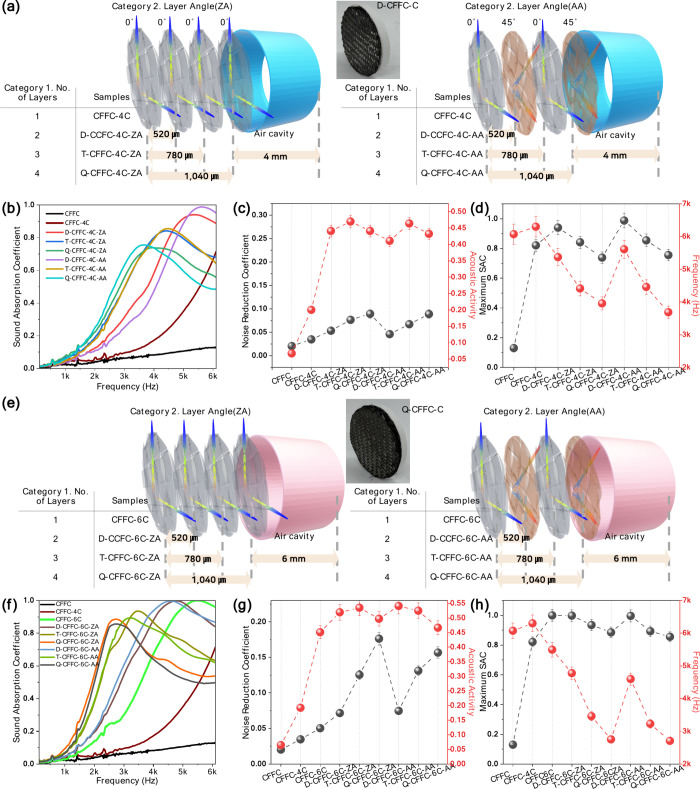
Sound absorption results for CFFC specimens with a single air cavity. Structural views for (a) 4 mm and (e) 6 mm cavities. Results of (b, f) SAC, (c, g) NRC and acoustic activity, and (d, h) maximum SAC values. The data demonstrate that cavity thickness directly governs resonance frequency and low frequency absorption efficiency, confirming that deeper cavities extend the absorption range through enhanced air structure coupling.

Building upon the single-cavity analysis, Figure 4 examines the acoustic performance of CFFC specimens incorporating multiple air cavities connected in series (CFFC-CxN). Two initial cavity heights, 4 and 6 mm, were investigated, and the number of cavity units (N = 1–4 for 4 mm and N = 1–3 for 6 mm) was varied to analyze cumulative resonance effects. Figures 4a and 4e show the stacking schemes for 4C and 6C units, respectively. In the 4 mm series, the configurations include CFFC-4C, CFFC-4Cx2, CFFC-4Cx3, and CFFC-4Cx4; in the 6 mm series, the corresponding configurations include CFFC-6C, CFFC-6Cx2, and CFFC-6Cx3. Each configuration is tested with both the ZA and AA orientations of the carbon fiber fabric. Here, CFFC-CxN refers to the number of single-cavity units arranged in series. In the 4 mm cavity series, multicavity specimens (CFFC-4CxN, N = 2–4) exhibit exceptionally high SAC values, typically ≥ 0.98, at their respective resonant frequencies, demonstrating highly efficient absorption within targeted bands. Notably, resonance shifts progressively downward as cavity count increases, ranging from ∼ 4,628 Hz for CFFC-4Cx2-AA to ∼ 2,460 Hz for CFFC-4Cx4-AA. NRC and acoustic activity also increase with cavity number (Figure 4c,d). For example, CFFC-4Cx4 achieves NRC ≈ 0.29 and acoustic activity ≈ 0.64, with minimal dependence on fiber orientation. A similar trend is observed for the 6 mm cavity series (Figures 4e–h). Specimens with two or more serial cavities consistently achieve SAC ≥ 0.97. Among these, CFFC-6Cx3-AA shows particularly strong performance (NRC ≈ 0.30, acoustic activity ≈ 0.65), despite a relatively compact overall height of ∼ 18.78 mm. A comparison between CFFC-6Cx2-AA (height ≈ 12.52 mm, NRC = 0.16) and CFFC-4Cx3-AA (height ≈ 12.78 mm, NRC = 0.14) indicates that specimens with similar total thickness produce comparable broadband absorption, suggesting that total structural height is an important parameter governing overall acoustic performance. The monotonic increases in NRC and acoustic activity with cavity count (Figures 4c and 4g) indicate that each cavity layer contributes an additional resonance. The resulting multi resonance behavior broadens the effective absorption bandwidth and enhances broadband performance, similar to multiresonator arrays. It is also noted that the role of the woven microstructure becomes more significant when the rear air cavity is introduced. While the fabric-only configuration provides mainly surface-level viscous dissipation, the cavity causes multiple rereflections inside the confined space. These rereflected waves interact with the interyarn pores and bundle undulation of the carbon fiber fabric, effectively creating microscale trapping pathways that enhance viscous and thermal losses, leading to the higher SAC peaks and broader absorption bandwidths observed in Figure 3. Stacking multiple air cavities in series therefore represents an effective and scalable strategy for achieving strong sound absorption over a wide frequency range. Overall, the acoustic behavior of the CFFC systems can be understood through a unified structure–property–performance relationship. Increasing the number of woven layers decreases the air permeability and increases the flow-path tortuosity, enhancing viscous and thermal dissipation. Fiber orientation modulates anisotropic shear deformation and frictional damping, particularly when coupled with cavity-induced oscillations. The introduction of a single or multiple air cavities modifies the effective acoustic reactance and lowers the resonance frequency, while serial cavity stacking produces multiresonant behavior that broadens the absorption bandwidth. These structural parameters collectively determine key performance indicators, such as SAC, NRC, and impact-noise attenuation, providing a consistent framework for tuning broadband absorption in lightweight CFFC-based composites.

**Figure 4:**
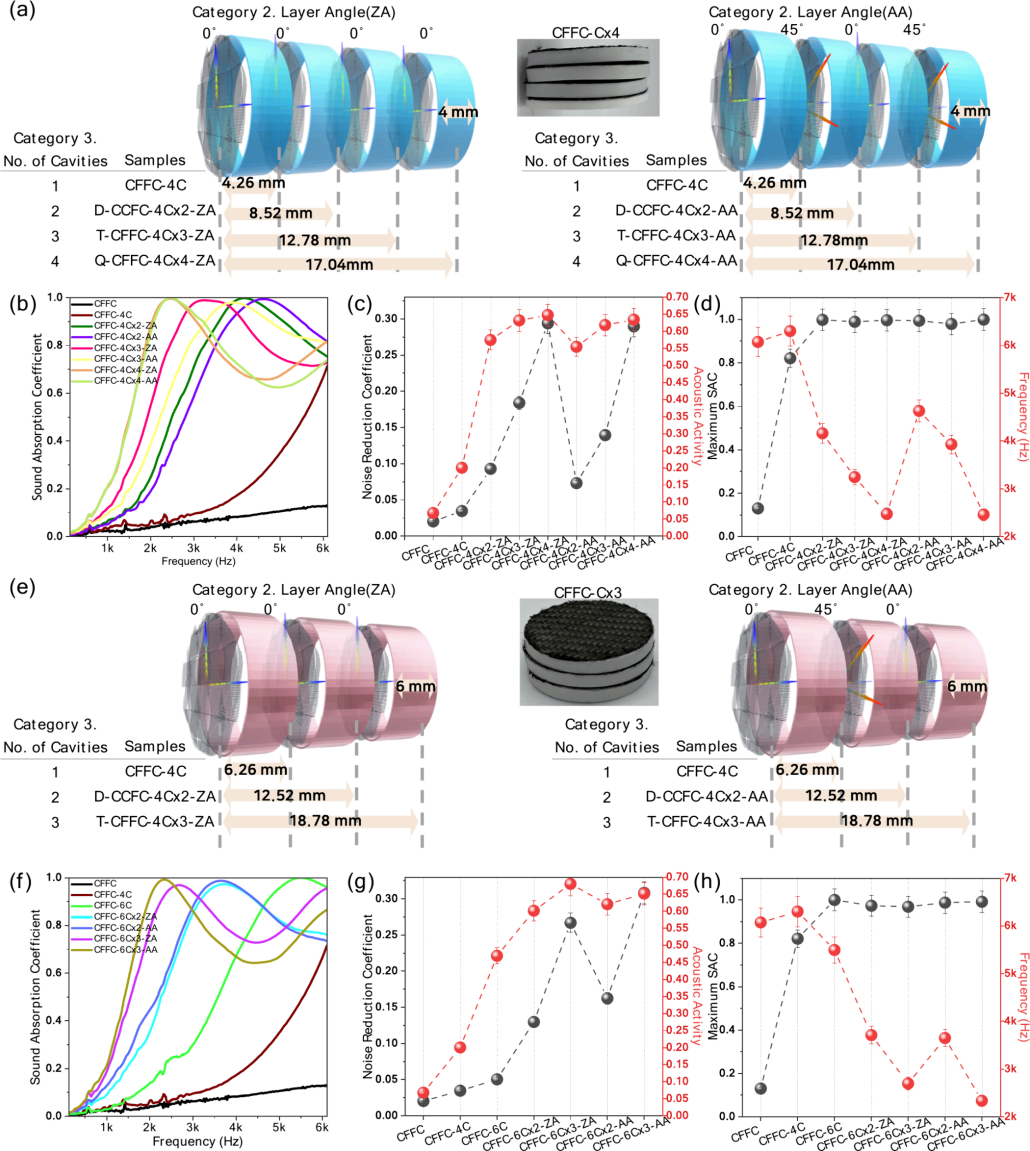
Sound absorption result of multiair cavity CFFC specimens. Structural configurations with one to four air cavities stacked in series for (a) 4 mm and (e) 6 mm cavities. Corresponding (b, f) SAC, (c, g) NRC and acoustic activity, and (d, h) maximum SAC values. As the number of serially connected cavities increases, multiresonant coupling broadens the effective absorption bandwidth and enhances overall NRC, demonstrating a scalable route for broadband acoustic optimization.

To evaluate the practical applicability of the developed CFFC absorbers under floor impact conditions, a simplified two-story acrylic house model (200 × 200 × 400 mm^3^) was constructed (Figure 5a). The walls and ceilings were made of 3 mm (3T) acrylic plates, and PLA cavity layers (4 or 6 mm) were 3D-printed in a 3 × 3 lattice pattern and installed beneath the second floor surface. Carbon fiber layers (1–4 plies, ZA or AA) were placed above the cavity layer. A 19.4 g steel weight was dropped from the upper floor, and the resulting sound pressure level (SPL) inside the first floor was measured by using a sound level meter. Figure 5c,d compares the SPL spectra of the bare house (BH) with those using CFFC-only damping layers. Across all ZA and AA configurations (CFFC, D-CFFC, T-CFFC, and Q-CFFC), similar SPL reduction patterns are observed. The maximum SPL reduction reaches 43.8 dB at 20 kHz for Q-CFFC-AA compared to BH. AA configurations generally outperform ZA configurations in the 10–100 Hz range, achieving up to ≈3 dB greater attenuation due to increased internal shear deformation and frictional dissipation. Figure 5e,f shows that introducing a 4 mm air cavity beneath the CFFC layer markedly improves impact noise reduction over a broader frequency range relative to cavity-free configurations. Double- and triple-layer CFFC-4C specimens (ZA and AA) exhibit progressively enhanced attenuation across 250–2,000 Hz, with T-CFFC-4C-AA achieving up to ∼ 30 dB SPL reduction relative to BH. Increasing cavity height to 6 mm further improves damping, especially in the low-to-mid frequency bands. Figure 5g compares CFFC-4C with D-CFFC-4C and T-CFFC-4C for the ZA orientation, showing incremental benefits proportional to layer count. Figure 5h highlights that D-CFFC-6C (two layers, 6 mm cavity) achieves attenuation comparable to that of Q-CFFC-4C (four layers, 4 mm cavity) in the low-frequency region (∼10–100 Hz), indicating that cavity depth can partially substitute for additional fabric layers. Across all cavity conditions, AA configurations show modestly higher attenuation at selected frequencies, but the overall trend indicates that layer count and cavity thickness are the dominant parameters in determining impact noise performance. The enhanced performance of cavity-integrated structures can be attributed to cavity-induced wave reflection and air column resonance, which, when combined with the woven fabric’s microstructural damping, increases energy dissipation. Multilayered CFFC structures further extend the acoustic transmission path, reducing SPL across a broad frequency range. The results confirm that integrating multilayer CFFC structures with tunable air cavities provides a lightweight and efficient strategy for attenuating impact noise in compact architectural systems.

**Figure 5:**
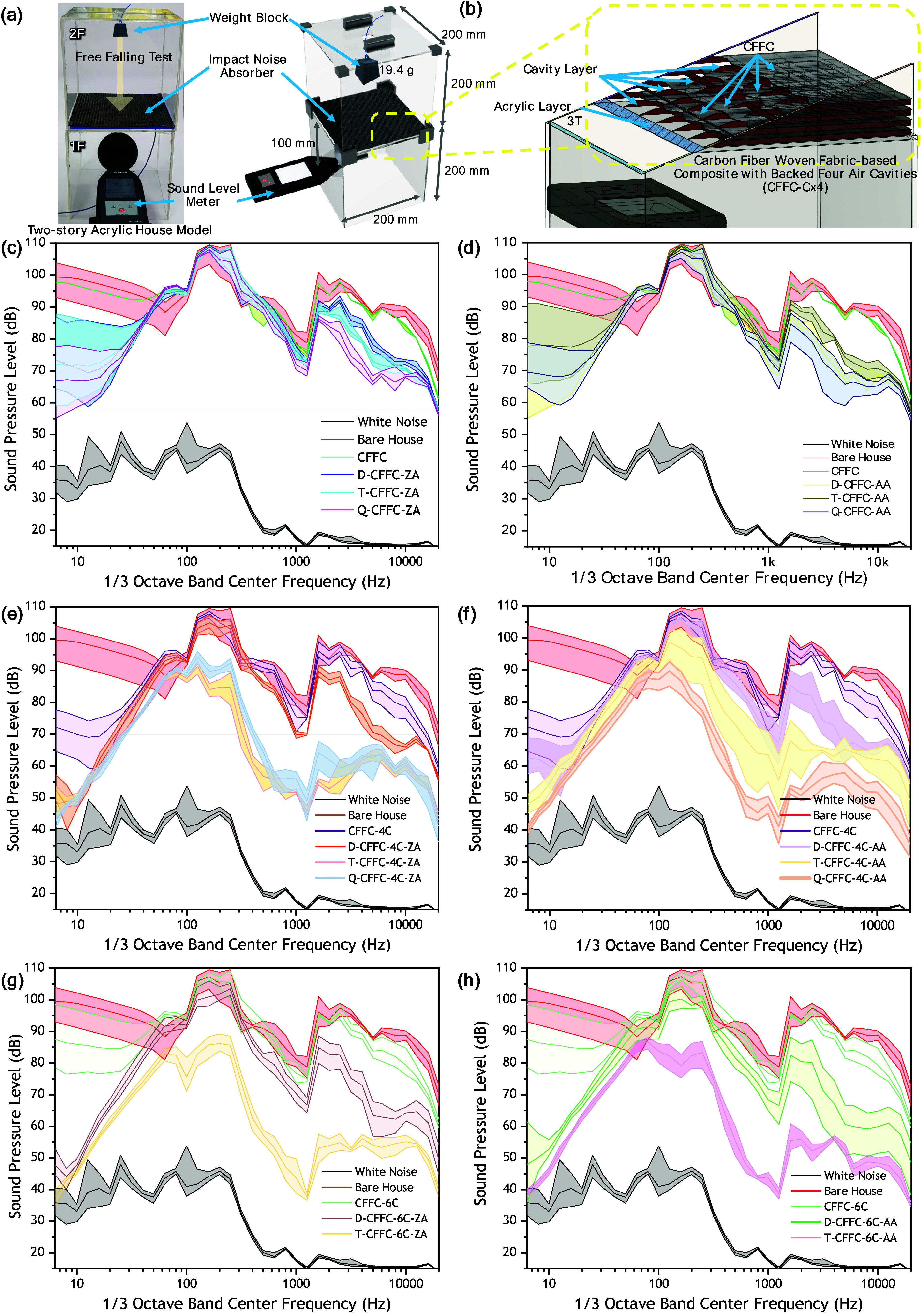
Impact sound test of a two-story acrylic house model. (a) Schematic of the impact noise attenuation setup employing CFFC absorbers. (b) Schematic cross section of CFFC-Cx4 structure. (c-h) Sound pressure level (SPL) spectra at 1/3 octave band center frequencies. The results validate that integrating multilayered carbon fiber fabrics with tunable air cavities significantly reduces transmitted sound, demonstrating the material’s real-world applicability for lightweight noise control systems.

## 4 Conclusion

This work establishes a comprehensive design framework for carbon-fiber woven fabric composites (CFFCs) in which acoustic performance emerges from the coupled effects of multilayer architecture, woven anisotropy, and cavity geometry. Layer stacking reduces air permeability and increases flow-path tortuosity, strengthening viscous and thermal dissipation within the fabric network. Fiber orientation further modulates anisotropic shear deformation and frictional damping, particularly when combined with rear-side cavity oscillations. Air cavities, either single or serially arranged, modify the effective acoustic reactance and shift the resonance toward lower frequencies, while multicavity configurations generate multiresonant behavior that broadens the absorption bandwidth. These structural parameters collectively dictate key performance indicators, such as SAC, NRC, and impact-noise attenuation. Experimental validation confirms the scalability and practicality of this design approach. Multilayer CFFC-C/CxN structures achieve high absorption levels within ultrathin profiles, and impact noise tests using a two-story acrylic model demonstrate substantial reductions in transmitted sound. The tunability offered by layer number, fiber orientation, and cavity configuration positions these composites as lightweight, sustainable candidates for next-generation architectural, transportation, and industrial noise control systems. Future work may further explore hybrid material integration, durability, and simulation-driven optimization to extend the design space of cavity-enabled acoustic composites.

## Supplementary Material



## Notes

Details of sample fabrication, additional acoustic measurements, and supplementary figures (PDF)
