# Supplementary material for: Eco-Friendly Carbon Fiber Woven Fabric-Based Sound Absorbers with Tunable Air Cavity Structures for Advanced Noise Control
Source: ACS Omega. 2026 Jan 27;11(5):8131–40. doi: 10.1021/acsomega.5c10560 (PMC12903152; doi:10.1021/acsomega.5c10560)
Supplement: Supplementary file 1 [file ao5c10560_si_001.pdf]

Supporting Information

Eco-Friendly Carbon Fiber Woven Fabric-Based Sound Absorbers with Tunable Air Cavity Structures for Advanced Noise Control

Jung-Hwan Oh , Yong-Won Kwon**

J.-H. Oh
National Center for Carbon Neutral Technology Strategy, Korea Institute of Energy Research(KIER), 152 Gajeong-ro, Yuseong-gu, Daejeon, 34129, Republic of Korea
Y.-W. Kwon
Department of Advanced Materials, Research Institute of Medium&Small Shipbuilding(RIMS), 38-6 Noksansandan 232-ro, Kangseo-gu, Busan, 46757, Republic of Korea

*Corresponding author.
E-mail address: jhoh@kier.re.kr (Jung-Hwan Oh), ywkwon@rims.re.kr(Yong-Won Kwon)

Figure S1 presents microscopic images of the fabricated ultra-thin carbon fiber woven fabric-based sound absorbers. The thickness of the carbon fiber woven fabric is 0.25 mm, and the diameter of an individual carbon fiber is approximately 50-100 ㎛. The thickness of the 3D-printed structural ring is 0.4 mm, and the diameter of an individual wire is about 100 micrometers.

Figure S2 shows the results of tensile tests on the carbon fiber woven fabric itself. As shown in Figure S2-a, tensile tests were conducted on a single layer of the carbon fiber woven

fabric. The results for a single layer of carbon fabric show a maximum stress of 265 MPa at a strain of 4%. The elastic modulus of the carbon fabric was approximately 0.44 GPa. After the tensile test, the carbon fibers did not break, but the fabric separated.

Figure S3 presents the impact sound transmission loss reduction achieved by various CFFC configurations, calculated as the difference in sound pressure level between the bare house (BH) condition and the measurements with CFFC absorbers. Panels (a) and (b) show specimens without an air cavity, panels (c) and (d) show specimens with a 4 mm air cavity, and panels (e) and (f) show specimens with a 6 mm air cavity. Results are shown for different numbers of fabric layers and for both zero-angle (ZA) and acute-angle (AA) stacking configurations.

Table S1 presents the weight and total height of the specimens used in the test according to the type of specimen and the height of the air cavity. The height of the air cavity hole is divided into 4 mm and 6 mm, and the diameter is divided into 30 mm and 60 mm, which are the diameters of the tubes used in the impedance test. The basic case refers to the carbon fiber woven fabric itself without an air cavity. In the case of a single air cavity, there is only one layer of air cavity with a height of 4 mm or 6 mm. In the case of multiple air cavities, the weight and total height according to the number of air cavity holes with a height of 4 mm or 6 mm are shown.

To ascertain the porous characteristics of the specimens belonging to categories 1 and 2, the air permeability of the CFFC specimens was measured as shown in Table S2. Three samples were prepared for each specimen, and the air permeability was measured for each sample, with the average value subsequently calculated. As the number of CFFC layers stacked in the ZA method increased from 1 to 4, the air permeability gradually decreased to 562 mm/s, 343 mm/s, 252 mm/s, and 182 mm/s, respectively. Similarly, when the number of CFFC layers was stacked from 2 to 4 in the AA method, the air permeability demonstrated a decreasing trend, exhibiting lower values compared to the ZA method.

Table S3 provides a comprehensive summary of the structural and acoustic parameters of all CFFC-based specimens examined in this study. The table compiles layer count, fiber orientation, cavity thickness, total thickness, and the corresponding acoustic performance metrics (NRC and SAC_{max}), presenting these values in a unified format for clear comparison across single layer, multilayer, cavity-backed, and multicavity configurations.

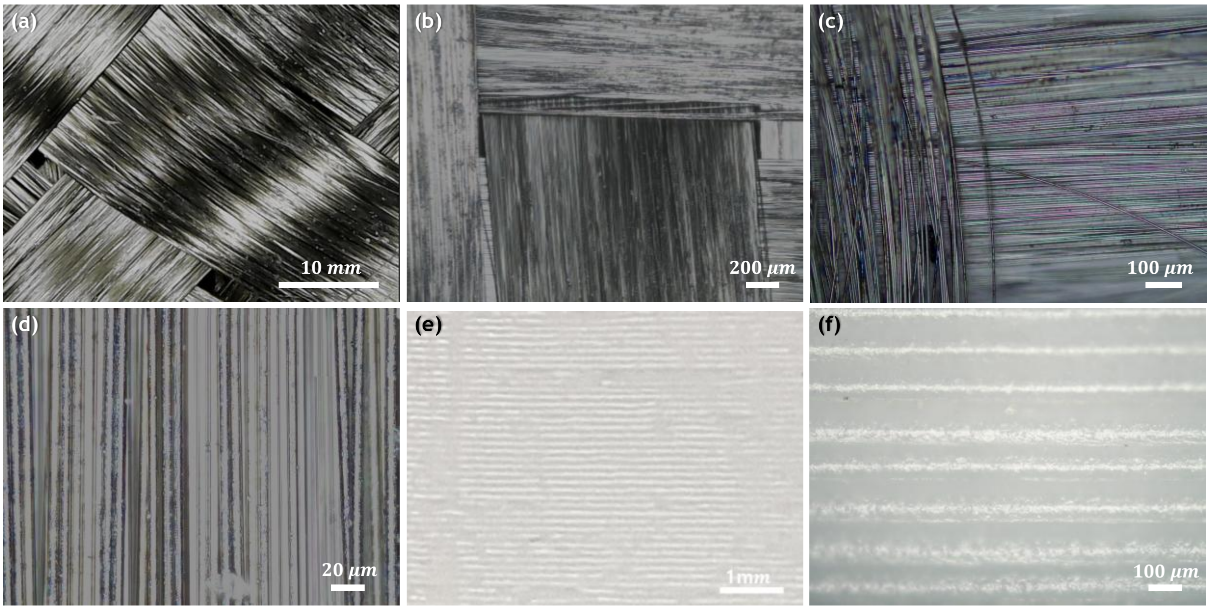

Figure S1. Optical microstructure of the carbon-fiber woven fabric. (a) Low-magnification view showing the twill weave pattern. (b) Interyarn pore openings (typical pore width ≈ 150–200 μm). (c) Bundle crossover illustrating periodic undulation of woven yarns. (d) Higher-magnification image showing filament-level surface texture and microscale dissipation pathways. (e-f) Surface image of 3D printed air cavity rings.

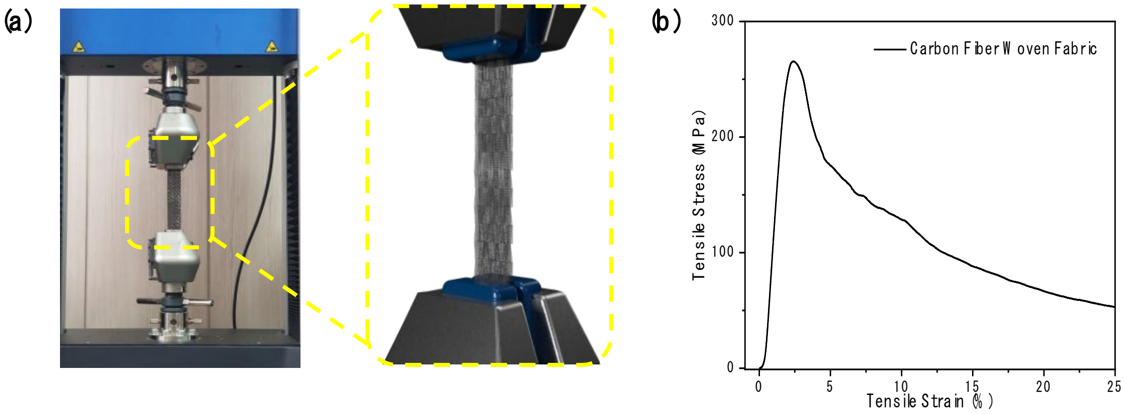

Figure S2. Mechanical Properties of Carbon Fiber Woven Fabrics: (a) UTM Test Setup, (b) Elastic Modulus.

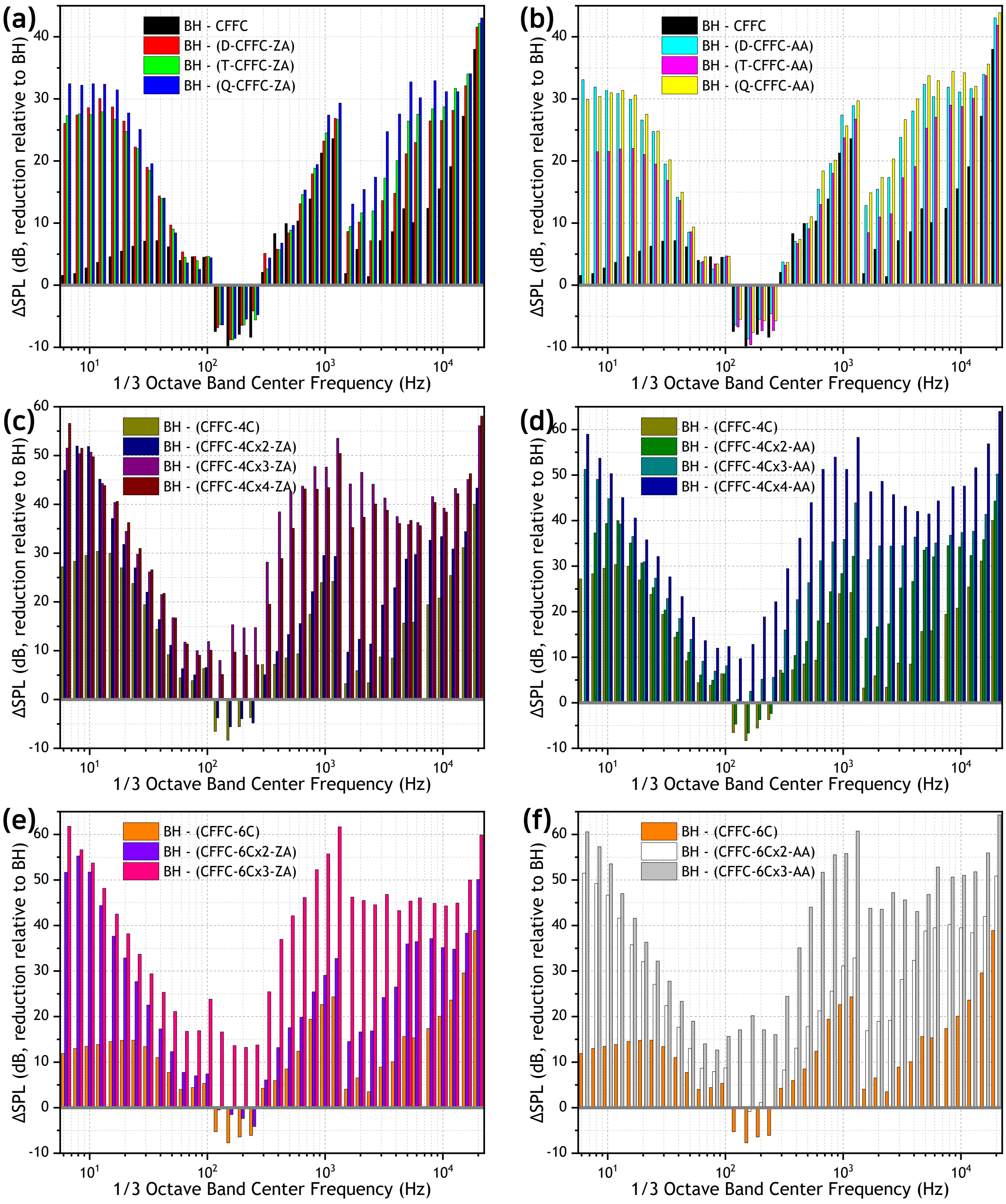

Figure S3. Difference in sound pressure level (ΔSPL) obtained from the impact-noise test, computed relative to the bare-house (BH) condition and plotted across 1/3-octave center frequencies on a logarithmic frequency scale. (a) ΔSPL for CFFC-ZA specimens. (b) ΔSPL for CFFC-AA specimens. (c) ΔSPL for CFFC-4C-ZA specimens. (d) ΔSPL for CFFC-4C-AA specimens. (e) ΔSPL for CFFC-6C-ZA specimens. (f) ΔSPL for CFFC-6C-AA specimens.

Table S1. Specifications of Test Specimens.

Case	Samples	D30			D60		
		Weight (g)	Thickness (mm)	Density (g/cm^3)	Weight (g)	Thickness (mm)	Density (g/cm^3)
Basic	CFFC	0.160	0.260	0.8706	0.330	0.260	0.4489
	D-CFFC	0.320	0.520	0.8706	0.660	0.520	0.4489
	T-CFFC	0.480	0.780	0.8706	0.990	0.780	0.4489
	Q-CFFC	0.640	1.040	0.8706	1.320	1.040	0.4489
Single Air Cavity	CFFC-4C	0.340	4.260	void-fraction-dominated	0.680	4.260	void-fraction-dominated
	D-CFFC-4C	0.500	4.520		1.010	4.520	
	T-CFFC-4C	0.660	4.780		1.340	4.780	
	Q-CFFC-4C	0.820	5.040		1.670	5.040	
	CFFC-6C	0.510	6.260		0.860	6.260	
	D-CFFC-6C	0.670	6.520		1.190	6.520	
	T-CFFC-6C	0.830	6.780		1.520	6.780	
	Q-CFFC-6C	0.990	7.040		1.850	7.040	
Multiple Air Cavities	CFFC-4C	0.340	4.260		0.680	4.260	
	CFFC-4Cx2	0.680	8.520		1.360	8.520	
	CFFC-4Cx3	1.020	12.780		2.040	12.780	
	CFFC-4Cx4	1.360	17.040		2.720	17.040	
	CFFC-6C	0.510	6.260		0.860	6.260	
	CFFC-6Cx2	1.020	12.520		1.720	12.520	
	CFFC-6Cx3	1.530	18.780		2.580	18.780	

Table S2. Table of air permeability of CFFC specimens.

Samples	Air permeability (mm/s)			
	1	2	3	Avg.
CFFC	584	540	563	562
D-CFFC-ZA	355	338	335	343
T-CFFC-ZA	264	245	247	252
Q-CFFC-ZA	182	184	179	182
D-CFFC-AA	328	294	306	309
T-CFFC-AA	240	216	226	227
Q-CFFC-AA	183	170	174	176

Table S3. Summary of structural and acoustic parameters for all CFFC-based specimens, including layer count, fiber orientation, cavity thickness, total thickness, and measured acoustic performance (NRC, SACmax). The table consolidates values extracted directly from experimental measurements in Figures 2–4 and Table S1, providing a unified reference for comparing single-layer, multilayer, cavity-backed, and multicavity configurations.

Specimen	Layer Count (N)	Orientation	Cavity Thickness (mm)	Cavity Units (N)	Total Thickness (mm)	NRC	SACmax
CFFC	1	-	0	0	0.26	2.03%	13.05%
D-CFFC-ZA	2	ZA	0	0	0.52	1.88%	20.63%
D-CFFC-AA	2	AA	0	0	0.52	2.47%	21.16%
T-CFFC-ZA	3	ZA	0	0	0.78	2.84%	45.50%
T-CFFC-AA	3	AA	0	0	0.78	3.04%	49.23%
Q-CFFC-ZA	4	ZA	0	0	1.04	3.79%	51.43%
Q-CFFC-AA	4	AA	0	0	1.04	3.67%	42.00%
CFFC-4C	1	-	4	1	4.26	3.47%	82.11%
D-CFFC-4C-ZA	2	ZA	4	1	4.52	5.32%	94.04%
D-CFFC-4C-AA	2	AA	4	1	4.52	4.58%	98.80%
T-CFFC-4C-ZA	3	ZA	4	1	4.78	7.64%	84.17%
T-CFFC-4C-AA	3	AA	4	1	4.78	6.71%	85.52%
Q-CFFC-4C-ZA	4	ZA	4	1	5.04	8.91%	73.89%
Q-CFFC-4C-AA	4	AA	4	1	5.04	8.88%	75.57%
CFFC-6C	1	-	6	1	6.26	5.04%	100.00%
D-CFFC-6C-ZA	2	ZA	6	1	6.52	7.18%	99.80%
D-CFFC-6C-AA	2	AA	6	1	6.52	7.48%	99.64%
T-CFFC-6C-ZA	3	ZA	6	1	6.78	12.55%	93.51%

T-CFFC-6C-AA	3	AA	6	1	6.78	13.13%	89.30%
Q-CFFC-6C-ZA	4	ZA	6	1	7.04	17.63%	88.56%
Q-CFFC-6C-AA	4	AA	6	1	7.04	15.68%	85.61%
CFFC-4Cx2-ZA	2	ZA	4	2	8.52	9.31%	99.81%
CFFC-4Cx2-AA	2	AA	4	2	8.52	7.32%	99.42%
CFFC-4Cx3-ZA	3	ZA	4	3	12.78	18.39%	98.91%
CFFC-4Cx3-AA	3	AA	4	3	12.78	13.94%	97.94%
CFFC-4Cx4-ZA	4	ZA	4	4	17.04	29.40%	99.62%
CFFC-4Cx4-AA	4	AA	4	4	17.04	28.98%	99.91%
CFFC-6Cx2-ZA	2	ZA	6	2	12.52	12.98%	97.30%
CFFC-6Cx2-AA	2	AA	6	2	12.52	16.24%	98.72%
CFFC-6Cx3-ZA	3	ZA	6	3	18.78	26.69%	96.88%
CFFC-6Cx3-AA	3	AA	6	3	18.78	30.83%	99.18%
